# An Assessment of West Nile and Usutu Viruses’ Seroprevalence in Hospitalized Patients: A Preliminary Study on Flavivirus Exposure in Eastern Romania

**DOI:** 10.3390/pathogens13020133

**Published:** 2024-01-31

**Authors:** Luciana Alexandra Crivei, Andrei Vata, Danut Teodor, Daniela Porea, Andreea Paula Cozma, Adriana Anita, Luanda Elena Oslobanu, Serban Morosan, Gheorghe Savuta

**Affiliations:** 1 Regional Center of Advanced Research for Emerging Diseases, Zoonoses and Food Safety, Department of Public Health, Faculty of Veterinary Medicine, Iași University of Life Sciences, 700490 Iași, Romania; daniela.porea@yahoo.com (D.P.); aeanita@uaiasi.ro (A.A.); luanda_elena@yahoo.com (L.E.O.); epirovet@yahoo.com (G.S.); 2 “Sf. Parascheva” Infectious Diseases Hospital of Iasi, 700490 Iasi, Romania; andreiandrei@yahoo.com (A.V.); teodordan@yahoo.com (D.T.); 3 Department of Infectious Diseases, Faculty of Medicine, “Grigore T. Popa” University of Medicine and Pharmacy Iasi, 700490 Iași, Romania; 4 Laboratories and Research Stations Department, Danube Delta National Institute for Research and Development, 820112 Tulcea, Romania; 5 Faculty of Veterinary Medicine, Iași University of Life Sciences, 700490 Iași, Romania; a.cozma@uaiasi.ro (A.P.C.); serban.morosan@sorbonne-universite.fr (S.M.); 6 Faculté de Médecine, Sorbonne Université, UMS 28, Inserm, 75013 Paris, France

**Keywords:** arbovirus, West Nile virus, Usutu virus, Romania, mosquito-borne diseases, seroprevalence, public health

## Abstract

WNV and USUV are closely related epornitic flaviviruses transmitted by *Culex* mosquitoes which can cause febrile and neurodegenerative disease in humans. The impact of both viruses on public health has increased in the recent decades. Aim: The aim of the study was to evaluate the seroprevalence of WNV and USUV in hospitalized patients from eastern Romania who did not show symptoms corresponding to the case definition. Methods: Human blood samples from the hospitalized patients were collected in 2015 and from April to September 2019 in Iasi County, Romania. The samples were screened by ELISA for anti-WNV IgG, IgM, and anti-USUV IgG antibodies. Results: A cumulative seroprevalence of 3.4% was recorded for anti-WNV IgG antibodies and 9.1% for anti-WNV IgM. No sample was positive for anti-USUV antibodies. Conclusion: The cumulative seroprevalence observed provides support for the consideration of WNV as being endemic in the east of Romania. The absence of anti-USUV antibodies may be related to cross-reactivity and cohort size, thus, USUV should be considered in clinical practice and become an objective for active surveillance in Romania.

## 1. Introduction

West Nile virus (WNV) and Usutu virus (USUV) are single-stranded and enveloped mosquito-borne viruses (MBVs) of the *Flaviviridae* family (genus *Flavivirus*) and are phylogenetically and antigenically related to the Japanese encephalitis virus complex, leading to misinterpretation and underestimation of the WNV and USUV infection burden [1]. For both viruses, mosquitoes from the *Culex* genus are their primary vectors, and, to a lesser extent, *Aedes* mosquitoes, while humans and animals are incidental hosts. However, humans and animals are considered to be “dead-end” hosts, as the viraemia in such hosts does not reach a level to further infect vectors during a blood meal [2]. While WNV represents a burden to animal and public health because of its ability to cause large epidemics [3], USUV has gained global attention due to its emergence, zoonotic potential, and ability to co-circulate with WNV [4]. Generally, in humans, WNV infection is asymptomatic (80%), while those with symptoms describe a flu-like syndrome (arthralgia, myalgia, and headache) [1]. Initially seen as a negligible human pathogen, lately, USUV infections have been noticed due to the associated neurological disorders in patients [5] and their many similarities to West Nile fever [1].

In Romania, WNV has been endemic since the mid-1990s, with large outbreaks in humans occurring in 1996, 2010, 2016 and 2018 [6,7]. Since the first large WNV epidemic in 1996, human infections have been reported yearly. Prior to 2016, the annual number of human cases did not exceed 52, whilst in 2016, 93 cases were documented. In 2018, the number of cases sharply increased (277 human cases) and, in the following year, the overall number of cases decreased to 67. In the subsequent seasons, a “low level” maintenance of virus transmission was associated with the reporting of six cases in 2020 and seven cases in 2021. USUV is less commonly detected in humans, although several studies have detected co-circulation with WNV [8]. In Romania, USUV was detected for the first time in *Cx.pipiens* vectors collected in 2019 [9], and indirect evidence of circulation was obtained in wild birds between 2018 and 2019 [10]. In humans, previous serosurvey studies have not detected USUV-specific antibodies.

In nature, arboviral infection relies on several factors connected to the three degrees of viral dissemination and amplification: vectors, amplifying hosts, and incidental hosts [11]. In humans, the occurrence of cases might be influenced by risk factors such as advanced age, immunosuppression, chronic renal disease, a history of cardiovascular disease, and the hepatitis C infection virus [11], as well as hypertension, renal disease, and diabetes [12]. In addition to the mentioned potential risk factors, the incidence of neuroinvasive illness and mortality was significantly associated with advanced age and male sex, as consequences of existing co-morbidities and behavioral factors [13].

The public health significance of WNV is illustrated by the risk of transmission through substances of human origin (SoHo) and intrauterine means. In addition, there is a risk associated with blood transfusions. To mitigate the risks of blood safety, various EU countries apply measures regulated by EU directives [14]. In contrast to WNV, clinical findings related to USUV are rarely reported and transfusion-transmitted infections have never been reported [15]. Considering the occurrence of WNV infections due to SoHo and the associated risk in asymptomatic blood donors, the safety of blood units has been investigated through seroprevalence studies conducted on cohorts in Romania [16], Hungary [1], Italy [17], Greece [18], and Germany [19].

Although studies on the seroprevalence of WNV infection in humans are routinely conducted in blood transfusion centers in some European countries, data on the prevalence of infection in hospitalized high-risk patients are still lacking. Therefore, it remains unclear whether there is a clear association between the aforementioned risk factors and silent circulation in asymptomatic patients. The purpose of this study was to assess a sero-epidemiological investigation of silent WNV and USUV circulation in hospitalized patients without any record of specific arboviral symptoms. Also, we aim to provide information on associations between other variables and asymptomatic infection with WNV or USUV after the 2018 WNV epidemic.

## 2. Materials and Methods

### 2.1. Human Sample Collection

For the purpose of this study, serum samples were collected from patients at the “Sf. Parascheva” Infectious Disease Hospital in Iasi from April to September 2019. The serums were stored at −20 °C until further use after collection.

During this research, a total of 104 serum samples were collected from hospitalized patients, who did not show symptoms corresponding to the case definition, i.e., any person over the age of 15 who exhibits fever, encephalitis, and meningoencephalitis or encephalitis during the transmission season (May to October) and who reports a history of mosquito bites [6]. At admission, patients who agreed to sampling were displaying clinical signs mostly associated with viral or bacterial infections, allergies of unknown origin, and cold- or flu-like symptoms.

Respondents who gave their consent for sampling filled out a questionnaire designed to collect information on demographics (sex and age), clinical information (pre-existing conditions), and living environment. In addition, the sampling protocol included questions about blood transfusions and foreign travel. Regarding medical history, the questionnaire consisted of questions concerning comorbidities such as diabetes, hypertension, cardiovascular, neurological, or renal conditions.

Additionally, 88 samples collected in 2015 and previously tested for hepatitis E virus (HEV) infection were assessed in the study [20]. Samples were collected from patients with various forms of hepatitis (mean age = 36.1, range 1–73).

### 2.2. Ethical Statement

This study was approved by the institutional review boards of the participating institutions (Ethics Commission of the Hospital for Infectious Diseases “Sf. Parascheva” Iasi)—Decision number 2101/07 February 2019 and Decision number 8/18 February 2015.

Prior to sample collection, all subjects agreed to participate and signed informed consent forms.

### 2.3. Serological Testing

A total of 88 human serum samples, collected in 2019, were screened for the detection of anti-WNV IgG and IgM antibodies for WNV and for anti-USUV IgG antibodies, using commercial ELISA kits (Euroimmun IgG and IgM West Nile—Medizinische Labordiagnostika AG, Lübeck, Germany and Euroimmun IgG Usutu—Medizinische Labordiagnostika AG, Lübeck, Germany). The additional 88 samples collected in 2015 were tested solely for the detection of specific IgG antibodies for WNV. Serological testing was performed following the manufacturer’s instructions and the specifications of each kit. Absorbance values were recorded at a 450 nm wavelength and the results were interpreted according to the manufacturer’s specifications.

### 2.4. Statistical Analyses

Since several factors may be linked with WNV infection, to test the significance of different variables (age group, sex, and living environment) and specific antibodies’ presence, a statistical analysis was performed using the Fisher’s Exact Test. *p*-values lower than 0.5 were considered to be significant. The 95% confidence interval (CI) calculation was performed using MS Excel 2019, predefining the calculation formula.

## 3. Results

### 3.1. Human Sample Collection

The study included 88 samples, collected in 2019, tested for the detection of specific IgM and IgG antibodies for WNV and IgG antibodies for USUV, using enzyme-linked immunosorbent assay (ELISA). Additionally, for a retrospective analysis, ELISA for WNV IgG antibody detection was performed on 88 more samples collected in 2015.

The mean patient age was 59 years (range 16–98). Of 104 samples collected, 88 were tested for the detection of specific antibodies (IgG and IgM) against WNV and USUV (IgG), which excluded sera from individuals who did not fall into the risk categories: pre-existing conditions or advanced age (Table 1).

### 3.2. Seroprevalence of WNV IgG and IgM Antibodies

Serological testing on humans sampled in 2019 showed the presence of IgG antibodies directed against WNV in 2 out of 88 samples [2.3%, 95% CI (−0.84–5.39)]. Additionally, 4 out of 88 serum samples collected in 2015 tested positive for WNV-IgG [4.5%, 95% CI (0.19–8.9)]. Overall, six samples tested positive for WNV IgG antibodies, showing a cumulative seroprevalence of 3.4% [95% CI (0.73–6.09)].

In eight samples [9.1%, 95%CI (3.08–15.1)], IgM antibodies were detected, indicating recent WNV infection. The serologic results are shown in Table 2.

### 3.3. Seroprevalence of USUV IgG Antibodies

USUV IgG antibodies were not detected in any of the serum samples.

### 3.4. Statistical Results

The *p* value for the tested variables (sex, age, living environment, or pre-existing comorbidities) was higher than 0.5, without any association between the variables, proving a nonsignificant trend (*p* < 0.5).

## 4. Discussion

In recent years, due to climate change and globalization, mosquito-borne flaviviruses have spread throughout Europe and pose an increasing threat to human and animal health [21]. Many similarities have been identified between USUV and WNV; they share geographical areas, as well as genetic and epidemiological characteristics. Because of all these similarities, co-infection of WNV and USUV is expected and detected in both humans and birds [21].

Following the 2018 WNV epidemic, we aimed to estimate the prevalence of WNV and USUV in hospitalized patients in Iasi County. We also checked the association between demographic and medical factors and WNV infection in the study cohort.

The overall WNV-IgG seroprevalence of 3.4% reported in this study shows the immune response after previous infections in two different years (after the WNV epidemic of 2018 and after 2015). It also supports the status of WNV as an endemic pathogen in the eastern side of Romania among people, either as a result of viral spread through viral persistence mechanisms or due to continuous viral reintroduction.

The WNV IgG seroprevalence determined from the 176 samples analyzed is similar to one found in Italy (3.7%) in 2507 individuals [22] and in Tunisia (4.3%) in 742 patients in 2003 [23], but lower than that in Algeria (9.8%) [24], Zambia (10.3%) [25], and Gabon (27.2%) [26]. In Romania, a previous study performed among asymptomatic blood donors in the north-western region of Romania showed an overall seroprevalence of 3.2% in a cohort of 1200 participants [16].

The two WNV IgG-positive samples (seroprevalence of 2.3%) collected in 2019 were from patients older than 50 years (81 and 56 years old). Regarding the comorbidities encountered, one of the samples (ID 44) was collected from a woman with cardiac complaints and the other from a febrile man with coughing. Interestingly, the four positive samples (seroprevalence of 4.5%) collected in 2015 were also positive for HEV infection [20]. Regarding the reason for admission, patients were previously diagnosed with acute non-A, non-B, and non-C hepatitis.

WNV IgM-positive samples were collected in April (n = 2), May (n = 4) and August (n = 2). Among the WNV IgM-positive samples (seroprevalence of 9.1%), three of eight patients had pneumonia, and the rest reported at least one concomitant disease, such as diabetes, hypertension, cardiac complications, and allergies caused by insect bites. Interestingly, an 81-year-old woman living in a rural settlement was positive for both WNV IgG and IgM antibodies (ID sample, 44), reflecting a significant and stable humoral response [27]. None of the positive patients reported a history of blood transfusion.

Concerning the absence of immunologic response of USUV, although in Europe, outbreaks of both viruses have increased numerically and geographically, suggesting viral endemicity rather than periodic reintroduction from endemic regions [28], in Romania, current and previous serologic findings in humans [16] are at odds with European epidemiologic patterns.

Although the absence of USUV-positive samples suggests that this virus was not present in humans in the study area, as the WNV-positive samples were USUV-negative, ruling out co-infection or cross-serological reaction, we cannot assume its absence, as this study was not supported by sentinel animal surveillance. Previously, we performed monitoring in dogs, and we found USUV IgG-positive samples in this species [29].

Regarding the significance of risk factors, the present study could not demonstrate that a particular pre-existing condition can influence seroconversion (*p*-values were not considered to be significant). However, considering the four IgG-positive results from the retrospective analysis of HEV-positive patients, viral hepatitis infections may represent a risk factor for WNV infection. Concerning the other risk factors mentioned in the literature, such as renal or cardiovascular disease, hypertension, or diabetes, our results could not demonstrate an association with existing conditions or statistically significant gender differences among patients.

This preliminary study, which focused on investigating the associations between medical conditions and asymptomatic carriers, has some limitations. The biggest limitation involves the absence of the gold standard assay, considering that most of the antibodies elicited by flaviviruses are directed against the immunodominant envelope protein (E), resulting in considerable serologic cross-reactivity. Such cross-reactivity often compromises diagnostic tests for both humans and animals, and therefore virus neutralization tests are the gold standard in flavivirus serology, although they require high containment facilities. In our study, the simultaneous testing of both viruses (WNV and USUV) and the absence of double positive results could exclude false positives, although the double testing of sera cannot exclude the gold standard method. Another limitation of the screening protocol that can lead to an underrepresentation of the seropositivity stems from the fact that the number of samples is not representative of the Iasi County population. The study population, however, represented only 0.011% (in both years) of the total population between 15 and over 60 years, although the serosurvey group was aimed to be based only on subjects developing other comorbidities that might be at risk. Finally, selective sampling approaches based on risk criteria (age, pre-existing conditions, and living environment) could be a limitation of our study, although these selection criteria are common in patients who clinically express infection. In perspective, we aim to conduct long-term epidemiological studies with larger cohorts selected stochastically and without discriminatory criteria to determine the real value of seroprevalence.

## 5. Conclusions

In summary, an examination of the serologic status of hospitalized patients may be a useful indicator for assessing exposure to mosquito-borne flavivirus infection. Further studies are needed to determine the true prevalence of MBV, as no USUV-positive sample was found in all the sera tested and WNV seroprevalence was low. In addition, we emphasize the need to implement surveillance measures on vectors and hosts, keeping in mind how the health of humans and animals are interconnected.

## Figures and Tables

**Table 1:** Demographic and clinical characteristics of the tested hospitalized patients.

Characteristic	n	%
**Demographic parameters**		
Age group (years)		
16–58	33	37.5
60–69	31	35.23
>70	24	27.27
Median age in years	63	
**Gender**		
Male	38	43.18
Female	50	56.82
**Living environment**		
Urban	26	29.55
Peri-urban	14	15.91
Rural	48	54.55
**Associated comorbidities**		
Hypertension	23	26.13
Diabetes	5	5.68
Cardiovascular diseases	5	5.68
Immunosuppression	1	1.13
Chronic renal diseases	12	13.63
Chronic liver diseases	21	23.86
Other conditions		
Digestive signs	10	11.36
Respiratory signs	6	6.81
Neurologic signs	13	14.77
Clinically healthy	3	3.4
Sepsis	7	7.95
Various infections	10	11.36

**Table 2:** Data regarding positive samples for WNV IgM and IgG antibodies.

Sample ID	Seropositivity	Sex	Age	Living Environment	Pre-Existing Medical Conditions
10	IgM	F	69	Urban	Diabetes, Hypertension, Pneumonia
12	IgM	M	34	Peri-urban	Pneumonia, Fever
20	IgM	F	61	Rural	Hepatitis C
38	IgM	F	65	Peri-urban	Other
40	IgM	F	70	Rural	Other
91	IgM	M	45	Rural	Allergic reaction/insect sting(bite)
44	IgM + IgG	F	81	Rural	Cardiovascular Diseases
92	IgM	M	64	Peri-urban	Pneumonia
55	IgG	M	56	Rural	Fever, Coughing
UN2015	IgG	M		-	Hepatitis E
MA2015	IgG	F	15	-	Hepatitis E
84344	IgG	-		-	Hepatitis E
83309	IgG	-		-	Hepatitis E

## Data Availability

All data generated or analyzed during this study are included in this published article. Other datasets used and/or analyzed can be made available by the corresponding author on reasonable request.
